# The impact of a school-based tooth-brushing program on dental caries: a cross-sectional study

**DOI:** 10.1186/s12199-019-0832-6

**Published:** 2019-12-30

**Authors:** Yuri Tashiro, Keiko Nakamura, Kaoruko Seino, Shiro Ochi, Hiroshi Ishii, Masaru Hasegawa, Yoshimichi Kawauchi, Mitsuyuki Chiba

**Affiliations:** 1 Department of Global Health Entrepreneurship, Division of Public Health, Graduate School of Medical and Dental Sciences, Tokyo Medical and Dental University, Yushima 1-5-45, Bunkyo-ku, Tokyo, 113-8519 Japan; 2 Ichikawa Dental Association, Chiba, Japan; 3 NGO Healthy City Support Organization, Tokyo, Japan

**Keywords:** Dental caries, School children, DMFT index, School environment, Zero-inflated negative binominal regression, Cross-sectional study

## Abstract

**Background:**

Promotion of oral health in children is recognized as one of the components of health-promoting schools (HPSs). However, few studies have addressed supportive school environments for children’s oral health. This study aimed to evaluate the status of dental caries in school children at HPSs, with the objective of examining the impact of a supportive school environment for oral health, considering the lifestyles of individual children and the socioeconomic characteristics of their communities.

**Methods:**

Data of 2043 5th-grade students in 21 elementary schools in Ichikawa city between 2008 and 2013 were analyzed. Children’s oral health status was evaluated using the decayed, missing, and filled permanent teeth (DMFT) index. A self-reported lifestyle questionnaire, a survey of the school environment promoting tooth-brushing, and community socioeconomic characteristics derived from the National Census data were included in the analyses. Bivariate analyses were conducted to evaluate the children’s DMFT status, and zero-inflated negative binominal (ZINB) regression was used to assess the relationships between DMFT and other variables.

**Results:**

Prevalence of dental caries in the permanent teeth of 5th-grade children (aged 10–11 years) was 33.3%, with a mean DMFT score (± SD) of 0.83 ± 1.50. According to multilevel ZINB regression analysis, children from schools with after-lunch tooth-brushing time showed a higher odds ratio (OR) for excess zero DMFT (OR = 1.47, 95% CI = 1.00–2.15, *P* = 0.049) as compared to those from schools without it. Neither bivariate analysis nor ZINB model analysis revealed any significant influence of children’s gender or use of a toothpaste with fluoride.

**Conclusions:**

The school-based environment supportive of oral health was significantly associated with a zero DMFT status in children. School-based efforts considering the socioeconomic characteristics of the area warrant attention even with declining prevalence of dental caries.

## Background

Poor oral health status in children is a growing public health concern worldwide, as it has been reported to correlate not only with the physical well-being of children as they grow into adults but also with their socioeconomic status (SES) [1, 2], and therefore, promotion of oral health, especially prevention of dental caries, from an early stage in life needs considerable attention.

Schools are known to be important avenues for children to acquire knowledge about health [3, 4]. A healthy school, or a health-promoting school (HPS), is a comprehensive school-based concept aimed at developing healthy lifestyle habits, preventing diseases and injuries, and promoting the health of children engaged in school activities through a multisectoral approach [3, 5, 6]. Promotion of oral health in children is recognized as one of the components of HPSs and the relevant frameworks have been applied worldwide [2, 4]. Although not many HPS programs focus on oral health, there are some school-based interventions for reducing dental caries of children [1, 2, 5, 7, 8].

Guidelines for HPSs of the World Health Organization (WHO) address the importance of developing a supportive environment in schools for promoting children’s health [2, 5, 9]. For the promotion of children’s health, individual factors influencing children’s oral health, such as their lifestyles and caretaker’s attitude, also need attention [10, 11]. Efforts have been oriented toward establishing policies for safe environments in schools to prevent injuries and secure safe water [5, 9]. However, few practices have yet addressed supportive school environments for promoting children’s oral health.

Besides these school-based programs, previous studies have explored the SES in children’s communities and their status of dental caries [12–16], indicating the importance of considering the socioeconomic characteristics of living areas for promoting oral health of children. However, only a few studies have analyzed the independent relationships between status of dental caries of Japanese children and school-based programs and socioeconomic characteristics of living areas.

This study aimed to evaluate the status of dental caries in school children at HPSs, with the objective of examining the impact of a supportive school environment for oral health, considering the lifestyles of individual children and the socioeconomic characteristics of their communities.

## Methods

### Health database

Ichikawa city, located in a suburb of Tokyo with a population of 490,000 as of 2017 [17], is characterized by communities with a diverse socioeconomic profile. The city launched its own healthy school program in 2005 including oral health programs [18]. As part of the HPSs in Ichikawa city, oral health checkups and questionnaire surveys among 5th-grade students in elementary public schools had been conducted in the selected 5–8 schools annually since 2008.

The program focuses on evaluating the status of dental caries in the permanent teeth of 5th-grade children (aged 10–11 years), which is in the late mixed dentition period for children getting permanent teeth. Children of 5th grade were expected to show greater participation rates than those of 6th grade, the final year of elementary school. Data from oral health checkups conducted from 2008 to 2013 and those from 21 schools were compiled. A questionnaire survey on the lifestyles of children was conducted in the same schools where oral health checkups were conducted. Clinical dentists delegated by the Ichikawa Dental Association examined children in each school. Dentists included in this study participated in a training using standardized visual materials before they conducted the dental examination of children. The status of dental caries was assessed through visual examination and probing, and recorded using the decayed, missing, and filled permanent teeth (DMFT) index. Decayed teeth were detected through untreated carious lesions with obvious cavitation, and missing teeth were counted if they were missing due to caries [19].

Oral health-related questions were selected from the children’s lifestyle questionnaire and answers to multiple-choice questions were dichotomized in the analysis. The answer to the first question on breakfast habit was categorized based on whether they eat breakfast or not (“eat alone” or “eat with family” as “Yes” and “often skip breakfast” or “usually do not eat breakfast” as “No”), and other questions were categorized based on the median value of answers to the questions: “Do you eat breakfast on every school day?” (Yes/No), “Do you always eat green and yellow vegetables?” (Yes/No), “How often do you eat sweet snacks (candy, chocolate, chewing gum, ice cream, etc.) in a week?” (≤ 2 days/≥ 3 days), “How often do you drink sweetened drinks (juice, liquid yogurt, sports drink, etc.) in a week?” (≤ 2 days/≥ 3 days), and “Do you use toothpaste containing fluoride for brushing?” (Yes/No or Not Sure).

Among 2573 eligible children, 2489 (96.7%) participated in the program and those with missing values were excluded from the analysis. Complete data of 2043 5th-grade children (79.4% of eligible children) were obtained with the cooperation of the Ichikawa Dental Association and used for the analysis.

### School environment survey

A questionnaire survey of elementary schools was conducted to determine the status of water supply facilities for their classrooms and whether they allocated tooth-brushing time in the school. Since public elementary schools in Japan offer school lunch to their students according to National law [20] and children usually spend their lunch time together in their classrooms, the existence of school-based after-lunch tooth-brushing time at the time of oral health checkups was used to assess the school environment for tooth-brushing. The results of the school survey were analyzed with the individual children’s database.

### Community characteristics based on the census data

To obtain an overview of the relationship between children’s SES and status of dental caries, their parents’ marital and education statuses are recognized as being among the factors that capture and explain the community characteristics [21–23]. To apply these variables to children’s data, the regional rate of single-parent families and university graduates was evaluated as the “community characteristics” in this study. These data were obtained from the National Census of 2010 [24, 25]. The rate of households with school-age (aged 6–18 years) child/children and a single-parent relative to the total number of households of school-age child/children was calculated as the “percentage of single-parent families in the community,” and the rate of people who had graduated from university/graduate schools per total number of people with any academic background among persons aged 15 years or older was calculated as the “percentage of university graduates in the community.” The census data were obtained at each street level and classified as per districts of the schools. These allocated school district-level data were then divided into quartiles (Q1–Q4) based on 21 school areas and included in the analysis so that the most deprived group (highest rate of single-parent families and lowest rate of university graduates) was included in the last quartile (Q4). Data of quartiles were compared with data of children from schools.

### Data analysis

Chi-square test was used to test the associations among the lifestyle questionnaire, community characteristics, and school environment pertaining to after-lunch tooth-brushing by children’s gender and presence of caries. As DMFT scores were not normally distributed (Shapiro–Wilk test: *P* < 0.001), mean DMFT scores were compared using Mann–Whitney *U* test and Kruskal–Wallis *H* test. Considering the skewed distribution of DMFT scores, Poisson, zero-inflated Poisson (ZIP), negative binominal, and zero-inflated negative binominal (ZINB) regressions were employed for modeling the count data of DMFT. Although Poisson and negative binominal regressions do not require the assumption of the normality of data, negative binominal regression is preferred over Poisson regression model, as negative binominal model can handle over-dispersed data whose variance scores largely exceed the mean score. Furthermore, zero-inflated models (i.e., ZIP and ZINB) account for data with excess zeros [26–28], and to choose the model with a better fit for the study, a likelihood-ratio test was adopted to compare ZINB and ZIP models, as ZIP model is nested in ZINB [27]. Moreover, Vuong’s test was conducted for a non-nested comparison of ZIP with Poisson and ZINB with a standard negative binominal model [28]. In addition, comparison of Akaike information criterion (AIC) followed as a measure of goodness-of-fit of the models with the preference for the lowest value. Finally, the ZINB regression model was adopted to assess the effects of independent variables on DMFT scores. The same independent variables were included in all the analyses, and children with missing data were excluded from the study. As children are nested within schools and their lifestyles may vary according to the schools that they attend, multilevel ZINB regression was also considered to identify whether the association between DMFT scores and other variables still exists after accounting for the differences between schools, with the school used as a grouping factor and use of a random intercept model. Data were analyzed using RStudio version 1.1.463 (RStudio Inc., Boston, MA, USA) and IBM SPSS Statistics for Windows, version 23.0. (SPSS Inc., Chicago, IL, USA), with the significance level set at 0.05.

## Results

Among the 2043 children aged 10–11 analyzed, the percentage of male children was 53.1%. The prevalence of dental caries in the permanent teeth was 33.3% (*n* = 680). DMFT scores ranged from 0 to 12, with a median score (10th percentile, 90th percentile) of 0 (0, 3) and a mean score (± SD) of 0.83 ± 1.50.

Table 1 presents distribution of lifestyles of the children classified by gender. The percentage of female children who reported a higher frequency of eating sweet snacks (≥ 3 days/week) was higher than that of male children (*P* = 0.025), and the percentage of female children who reported a higher frequency of drinking sugar-sweetened drinks (≥ 3 days/week) was lower than that of male children (*P* = 0.011). All the schools were equipped with water supply facilities (water taps) in their classrooms, which the children could use for brushing their teeth. Seven schools reported having allocated a specific time after-lunch tooth-brushing time for the children. Integration of the results of the school environment survey with the individual children’s database showed that 27.8% children attended schools that had allocated a specific time for after-lunch tooth-brushing (Table 1). The percentage of single-parent families by school areas ranged from 7.54 to 16.17% and the percentage of university graduates ranged from 12.83 to 38.48%, in 21 school areas, respectively.

**Table 1:** Lifestyle behaviors, school environment for after-lunch tooth-brushing, and community characteristics of children, 2008–2013 (*n* = 2043)

	Total		Male		Female		*P*^1^
	*n*	%	*n*	%	*n*	%	
All Children	2043	100%	1084	53.1%	959	46.9%	
*Lifestyle behaviors*							
Do you eat breakfast on every school day?							
Yes	1954	95.6%	1033	95.3%	921	96.0%	0.412
No	89	4.4%	51	4.7%	38	4.0%	
Do you always eat green and yellow vegetables?							
Yes	901	44.1%	462	42.6%	439	45.8%	0.151
No	1142	55.9%	622	57.4%	520	54.2%	
How often do you eat sweet snacks in a week?							
≤ 2 days	972	47.6%	541	49.9%	431	44.9%	0.025*
≥ 3 days	1071	52.4%	543	50.1%	528	55.1%	
How often do you drink sweetened drinks in a week?							
≤ 2 days	1145	56.0%	579	53.4%	566	59.0%	0.011*
≥ 3 days	898	44.0%	505	46.6%	393	41.0%	
Do you use toothpaste containing fluoride for brushing?							
Yes	948	46.4%	497	45.8%	451	47.0%	0.594
No/Not Sure	1095	53.6%	587	54.2%	508	53.0%	
*School environment*							
School has after-lunch tooth-brushing time							
Yes	568	27.8%	301	27.8%	267	27.8%	0.970
No	1475	72.2%	783	72.2%	692	72.2%	
*Community characteristics*							
Percentage of single-parent families in community (%)							
Q1 (7.54–9.59)	508	24.9%	274	25.3%	234	24.4%	0.692
Q2 (9.94–10.99)	552	27.0%	283	26.1%	269	28.1%	
Q3 (11.00–11.78)	424	20.8%	222	20.5%	202	21.1%	
Q4 (11.86–16.17)	559	27.4%	305	28.1%	254	26.5%	
Percentage of university graduates in community (%)							
Q1 (28.21–38.48)	470	23.0%	259	23.9%	211	22.0%	0.515
Q2 (25.14–26.98)	615	30.1%	312	28.8%	303	31.6%	
Q3 (22.70–24.13)	437	21.4%	232	21.4%	205	21.4%	
Q4 (12.83–21.11)	521	25.5%	281	25.9%	240	25.0%	

*Q* quartile

^1^Chi-square test by gender

*P < 0.05 (two-tailed)

**Fig. 1:**
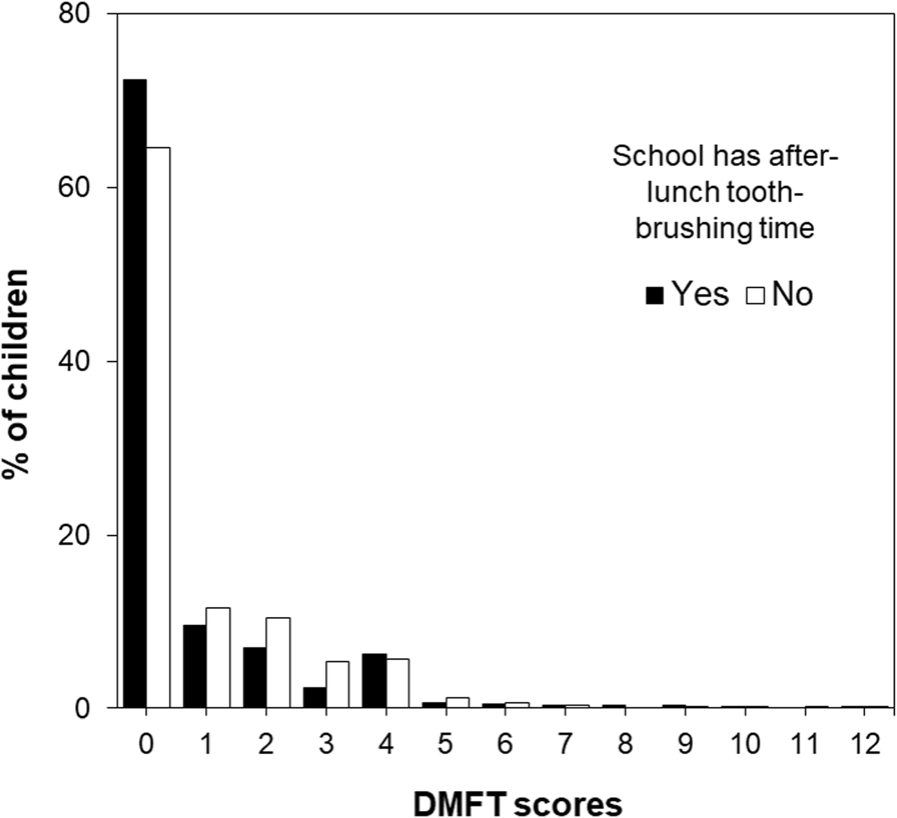
Distribution of DMFT scores according to whether the schools had allocated after-lunch tooth-brushing time (*n* = 2043)

Figure 1 shows the skewed distribution of DMFT scores according to whether the school had allocated after-lunch tooth-brushing time. The percentage of zero DMFT children among those who attended schools that were and were not allocated after-lunch tooth-brushing time was 72.4% and 64.5%, respectively.

Table 2 presents the DMFT status and mean DMFT scores classified by gender, lifestyles, school environment, and community characteristics of children. In addition, mean DMFT scores in children with DMFT > 0 were calculated. The percentage of children attending schools that had allocated after-lunch tooth-brushing time was higher in the children with DMFT = 0 (30.2%) than those with DMFT > 0 (23.1%) (*P* < 0.001). Mean DMFT score in children attending schools with after-lunch tooth-brushing time (0.74 ± 1.58) was lower than that in children attending schools without it (0.86 ± 1.47) (*P* = 0.002).

Table 3 shows model fit statistics of the four regression models. According to the results of likelihood-ratio test (ZINB vs. ZIP, *P* < 0.0001), Vuong’s test (ZIP vs. Poisson, *P* < 0.0001 and ZINB vs. negative binominal, *P* < 0.0001), and comparison of AIC, a better goodness-of-fit was suggested for ZINB in this study.

**Table 3:** Model fit statistics of regression models

	Log likelihood	AIC
Poisson	− 2865.93	5759.86
ZIP	− 2339.21	4734.42
Negative binominal	− 2398.00	4826.00
ZINB	− 2326.18	4710.35

*AIC* Akaike information criterion, *ZIP* zero-inflated Poisson, *ZINB* zero-inflated negative binominal (single level)

**Table 2:** DMFT status in children, 2008–2013 (*n* = 2043)

	DMFT = 0		DMFT > 0		*P*^*2*^	Mean DMFT (± SD)	*P*^*3*^	Mean DMFT among children with DMFT > 0 (± SD)	*P*^*3*^
	*n*	%	*n*	%					
All children	1363	66.7%	680	33.3%		0.83 ± 1.50		2.49 ± 1.64	
Gender									
Male	728	53.4%	356	52.4%	0.651	0.78 ± 1.40	0.492	2.39 ± 1.47	0.267
Female	635	46.6%	324	47.6%		0.88 ± 1.61		2.59 ± 1.80	
*Lifestyle behaviors*									
Do you eat breakfast on every school day?									
Yes	1312	96.3%	642	94.4%	0.054	0.82 ± 1.50	0.042*	2.48 ± 1.65	0.533
No	51	3.7%	38	5.6%		1.08 ± 1.55		2.53 ± 1.39	
Do you always eat green and yellow vegetables?									
Yes	625	45.9%	276	40.6%	0.024*	0.78 ± 1.52	0.027*	2.53 ± 1.78	0.961
No	738	54.1%	404	59.4%		0.87 ± 1.49		2.45 ± 1.54	
How often do you eat sweet snacks in a week?									
≤ 2 days	649	47.6%	323	47.5%	0.961	0.77 ± 1.33	0.642	2.30 ± 1.35	0.060
≥ 3 days	714	52.4%	357	52.5%		0.88 ± 1.64		2.65 ± 1.85	
How often do you drink sweetened drinks in a week?									
≤ 2 days	782	57.4%	363	53.4%	0.087	0.79 ± 1.47	0.102	2.48 ± 1.62	0.878
≥ 3 days	581	42.6%	317	46.6%		0.88 ± 1.55		2.49 ± 1.66	
Do you use toothpaste containing fluoride for brushing?									
Yes	633	46.4%	315	46.3%	0.960	0.83 ± 1.51	0.973	2.50 ± 1.64	0.707
No/Not Sure	730	53.6%	365	53.7%		0.82 ± 1.50		2.47 ± 1.63	
*School environment*									
School has after-lunch tooth-brushing time									
Yes	411	30.2%	157	23.1%	< 0.001**	0.74 ± 1.58	0.002**	2.68 ± 1.97	0.480
No	952	69.8%	523	76.9%		0.86 ± 1.47		2.43 ± 1.52	
*Community characteristics*									
Percentage of single-parent families in community (%)									
Q1 (7.54–9.59)	384	28.2%	124	18.2%	< 0.001**	0.46 ± 0.94	< 0.001**^✝^	1.89 ± 0.96	< 0.001**^✝^
Q2 (9.94–10.99)	348	25.5%	204	30.0%		0.95 ± 1.64		2.58 ± 1.75	
Q3 (11.00–11.78)	327	24.0%	97	14.3%		0.50 ± 1.13		2.21 ± 1.37	
Q4 (11.86–16.17)	304	22.3%	255	37.5%		1.28 ± 1.85		2.80 ± 1.80	
Percentage of university graduates in community (%)									
Q1 (28.21–38.48)	363	26.6%	107	15.7%	< 0.001**	0.45 ± 1.01	< 0.001**^✝^	1.99 ± 1.19	< 0.001**^✝^
Q2 (25.14–26.98)	359	26.3%	256	37.6%		1.09 ± 1.69		2.63 ± 1.67	
Q3 (22.70–24.13)	326	23.9%	111	16.3%		0.55 ± 1.17		2.16 ± 1.39	
Q4 (12.83–21.11)	315	23.1%	206	30.3%		1.08 ± 1.77		2.74 ± 1.83	

*DMFT* decayed, missing, and filled permanent teeth, *Q* quartile, *SD* standard deviation

^2^Chi-square test by DMFT existence

^3^Mann–Whitney *U* test for mean DMFT score by gender and questionnaire answer patterns

^✝^Kruskal–Wallis *H* test for mean DMFT scores by community characteristics quartiles

*P < 0.05 (two-tailed)

**P < 0.01 (two-tailed)

**Table 4:** ZINB regression models of associations between independent variables and children’s DMFT status (*n* = 2043)

	Zero-inflated model				Count model			
	Single level		Multilevel		Single level		Multilevel	
	OR^a^	95% CI	OR^a^	95% CI	IRR^b^	95% CI	IRR^b^	95% CI
			Fixed effects				Fixed effects	
Gender (female vs. male)	0.99	0.78–1.26	1.01	0.79–1.29	1.08	0.94–1.24	1.09	0.95–1.26
*Lifestyle behaviors*								
Do you eat breakfast on every school day? (Yes vs. No)	1.58	0.83–3.01	1.50	0.81–2.79	0.89	0.65–1.23	0.86	0.63–1.17
Do you always eat green and yellow vegetables? (Yes vs. No)	1.22	0.95–1.55	1.23	0.96–1.57	1.09	0.94–1.25	1.09	0.95–1.25
How often do you eat sweet snacks in a week? (≤ 2 days vs. ≥ 3 days)	0.87	0.68–1.13	0.89	0.69–1.15	0.80**	0.69–0.93	0.84*	0.72–0.97
How often do you drink sweetened drinks in a week? (≤ 2 days vs. ≥ 3 days)	1.27	0.98–1.64	1.27	0.98–1.65	1.08	0.93–1.25	1.08	0.94–1.25
Do you use toothpaste containing fluoride for brushing? (Yes vs. No/Not Sure)	0.99	0.77–1.26	0.94	0.73–1.21	1.03	0.89–1.19	0.98	0.85–1.13
*School environment*								
School has after-lunch tooth-brushing time (Yes vs. No)	1.63**	1.19–2.21	1.47*	1.00–2.15	1.28**	1.07–1.55	1.06	0.78–1.46
*Community characteristics*								
Percentage of single-parent families in community (%)								
Q1 (7.54–9.59)	0.91	0.57–1.47	0.94	0.54–1.63	0.51**	0.37–0.71	0.58*	0.37–0.91
Q2 (9.94–10.99)	1.02	0.70–1.47	1.05	0.67–1.67	0.79*	0.64–0.96	0.80	0.55–1.16
Q3 (11.00–11.78)	2.33**	1.61–3.37	2.39**	1.49–3.82	0.69*	0.54–0.87	0.77	0.52–1.14
Q4 (11.86–16.17)	Ref		Ref		Ref		Ref	
Percentage of university graduates in community (%)								
Q1 (28.21–38.48)	2.66**	1.69–4.19	2.67**	1.54–4.62	0.89	0.65–1.22	0.86	0.55–1.36
Q2 (25.14–26.98)	0.97	0.70–1.33	0.93	0.61–1.42	0.92	0.78–1.09	0.91	0.63–1.32
Q3 (22.70–24.13)	1.75**	1.19–2.57	1.64*	1.00–2.68	0.77*	0.61–0.98	0.75	0.49–1.14
Q4 (12.83–21.11)	Ref		Ref		Ref		Ref	

*ZINB* zero-inflated negative binominal, *DMFT* decayed, missing, and filled permanent teeth, *CI* confidence interval, *Q* quartile

^a^Odds ratio for being an excess zero DMFT (0 or ≥ 1) of the group in interest against reference group, holding other variables constant

^b^Incidence rate ratio for change in mean DMFT (≥ 1) of the group in interest against reference group, holding other variables constant

*P < 0.05

**P < 0.01

Table 4 summarizes the results of adjusted odds ratio (OR) and incidence rate ratio (IRR) obtained using the ZINB regression model. The ZINB regression combined the model of subjects with zero count into the logit model and subjects with over zero count into the count (negative binominal) model, which enabled the estimation of the risk of caries prevalence (DMFT = 0 or not) in OR and caries severity (increase in mean DMFT score) in IRR simultaneously [26–28]. For the multilevel ZINB model, intercept variances of the random effects of the zero-inflated model and count model part were 0.036 and 0.056, respectively. Children attending schools that had allocated time for after-lunch tooth-brushing showed higher odds of an excess zero DMFT status (OR = 1.63, 95% CI = 1.19–2.21, *P* = 0.002 in the single-level model and OR = 1.47, 95% CI = 1.00–2.15, *P* = 0.049 in the multilevel model). The school environment showed an inverse association with DMFT score in the single-level count model, which was not seen in the multilevel model. In the count model part, a lower consumption of sweet snacks per week (≤ 2 days) was associated with a decreased mean DMFT score (IRR = 0.80, 95% CI = 0.69–0.93, *P* = 0.004 in the single-level model and IRR = 0.84, 95% CI = 0.72–0.97, *P* = 0.019 in the multilevel model) as compared to a higher frequency of consumption of sweet snacks (≥ 3 days). With regard to the community characteristics, Q3 for single-parent family quartiles and Q1 and Q3 for university graduate quartiles showed higher odds of an excess zero DMFT status both in single-level and multilevel models. Conversely, Q1, Q2, and Q3 for both single-parent family and university graduate quartiles showed decreased mean DMFT scores in the count model as compared to the reference group, although the results were not statistically significant in all cases. Children’s gender and use of a toothpaste with fluoride had no significant influence, as analyzed using either bivariate analysis or the ZINB model.

## Discussion

This study described the status of dental caries in the permanent teeth of 5th-grade students attending HPSs and analyzed its relationships with their lifestyle behaviors, supportive school environments, and community characteristics. Some lifestyle behaviors showed a relationship with the status of dental caries, and school-based environment for tooth-brushing after lunch was associated with a higher OR of children with zero DMFT.

The school environment survey revealed that all the schools had water taps in each of their classrooms, and this study focused on Japanese schools that aimed to improve their environmental hygiene as one of their healthy school strategies. A physical environment (water taps in every classroom) is an important component as a tangible aspect of the HPS program. This study investigated both the tangible (water taps) and intangible (tooth-brushing time) aspects of HPSs and showed their relationship with the status of dental caries in children. Even in some schools that had not allocated school-wide brushing time, they had other programs such as school-wide mouth rinsing or classroom-based tooth-brushing after lunch. A positive attitude toward oral health promotion is already quite well established among schools in Ichikawa city, and this study adds further evidence of the school-based environment to encourage brushing to improve children’s oral health status.

The associations between the status of dental caries in children and the community-level SES were addressed in this study. Associations between SES characteristics and prevalence of dental caries at the prefectural level have already been reported in Japan [29, 30]. However, dental caries by school- or local district-level SES had been scarcely addressed in Japan. This study aimed to enable stakeholders of schools or local municipalities to consider more focused implementation of children’s oral health programs according to each school’s background and their areal socioeconomic environment.

No significant influence of children’s gender on DMFT status was identified in any of the analyses, which is consistent with previous reports [31, 32]. In addition, no significant influence was identified in the use of a toothpaste with fluoride. A previous review showed that the use of a fluoride toothpaste for caries prevention was only significant for toothpastes carrying maximum or higher fluoride concentrations than those approved in Japan at the time of the study [33]. Few Japanese studies have shown an association between topical fluoride application and caries status in children [10, 34]; however, artificial tap water fluoridation and mass topical fluoride application were not implemented in Ichikawa city at the time of the study.

The national average of caries prevalence of school children in Japan has been declining since the last 30 years [35, 36], and in particular, in 5th-grade children (i.e., from 64.1 to 53.9% between 2008 and 2013) [37]. Caries prevalence in the children in this study might have decreased irrespective of the tooth-brushing program in elementary schools. However, the children in this study had a lower caries prevalence than that reported in other studies in Japan [38–40].

This study showed higher mean DMFT scores among children with DMFT > 0 in schools that had after-lunch tooth-brushing time than in those without it; single-level ZINB regression predicted a higher IRR of mean DMFT scores among children in schools with after-lunch tooth-brushing time than those in schools without it. There were only 10 children with DMFT scores over 7 (including 6 children from schools with after-lunch tooth-brushing time and 4 children from schools with no such allocation) between 2008 and 2013 (Fig. 1). Some schools might have implemented the tooth-brushing program based on the high prevalence or severity of dental caries in children in their school. When a longitudinal study to evaluate influence of school-based environment and programs is conducted, adjustment by the status of dental caries at the baseline or standardizing by the prevalence of dental caries by school at baseline is required.

There are several strengths in this study. The study examined the permanent teeth of children in the 5th grade, which is in late mixed dentition period. This allowed the construction of a discussion on the relationship between school environment or programs and the status of dental caries, because permanent teeth generally appear from the age of 6 years, which is the age that children begin to attend school. The association between the status of dental caries of permanent teeth and school environment exposed by the children is worthy for discussion. Data were collected from multiple sources and combined them for a comprehensive analysis, based on long-term experience of HPSs with activities for promotion of oral health. The findings of this study serve as useful information for other communities focused on the promotion of children’s oral health. Another strength of this study is the use of the ZINB regression. When modeling count data with many zeros such as DMFT, a zero-inflated model should be considered to avoid the estimation parameters [27, 28]. The ZINB regression was shown to be the best fit for this study to obtain informative outcomes with regard to the children’s oral health status.

There are some limitations in this study and the results should be interpreted carefully. The number of permanent teeth among the subjects who were aged 10–11 is smaller than that of older age children; therefore, the prevalence of dental caries of permanent teeth presented in this study is age specific. The results of DMFT status should be interpreted carefully to generalize to children of all ages. The analysis is a cross-sectional design, although data derives over 6 years; therefore, the results are not able to address causal relationships. To clearly address the effectiveness of school-based oral health programs, a longitudinal design is recommended. Oral health checkups and lifestyle questionnaire surveys were conducted in randomly selected schools every year, and therefore, systematic chronological analysis of data by schools was not possible. Data on community characteristics were obtained from the National Census and allocated to school district-level to represent socioeconomic characteristics by school areas; therefore, socioeconomic indicators in this analysis represent characteristics by school areas but not by individual child.

This study highlighted the existence of children with severe DMFT scores, as a previous study from Japan raised this concern [38]. In addition, this study revealed the relationship between lifestyle behaviors, such as skipping of breakfast, and the status of caries in children, although no significant association was shown in the regression analysis. As a severe status of caries and lifestyles at home often reflect other social aspects or family environment [41, 42], further studies considering the overall lifestyles of children and attitudes of caretakers [11] are required.

## Conclusion

The findings of this study indicated that even among children in Japan, where the prevalence of dental caries has already declined, the school-based environment supportive of tooth-brushing was associated with a zero DMFT status in children. Higher SES of the communities, where the school is located, were also associated with a zero DMFT status in children. Further school-based efforts considering the socioeconomic characteristics of the area warrant attention for promoting children’s oral health.

## Data Availability

The dataset of children subjected in the present study is not publicly available due to ethical restrictions. The datasets derived from the National Census, statistics report from Ichikawa city, and reports from the Ministry of Education, Culture, Sports, Science, and Technology are available from respective sources.

## Abbreviations

AIC: Akaike information criterion
CI: Confidence interval
DMFT: Decayed, missing, and filled permanent teeth
HPS: Health-promoting school
IRR: Incidence rate ratio
OR: Odds ratio
SD: Standard deviation
SE: Standard error
SES: Socioeconomic status
WHO: World Health Organization
ZINB: Zero-inflated negative binominal
ZIP: Zero-inflated Poisson
